# Hypoxia Triggers ALYREF‐Mediated m^5^C Methylation of KIF20A to Activate KIF20A/BUB1 for Generating Ferroptosis Resistance in Cervical Cancer Cells

**DOI:** 10.1002/kjm2.70093

**Published:** 2025-09-07

**Authors:** Yan Gao, Ting Zou

**Affiliations:** ^1^ Department of Gynecology Guizhou Provincial People's Hospital Guiyang Guizhou China

**Keywords:** ALYREF, cervical cancer, ferroptosis resistance, hypoxia, KIF20A

## Abstract

Ferroptosis resistance is a key player in cervical cancer (CC) development. Hypoxia is a negative factor affecting CC treatment by inducing ferroptosis resistance. Our study aimed to investigate the detailed mechanisms of hypoxia‐induced ferroptosis resistance in CC cells. The mRNA and protein levels were assessed using RT‐qPCR and western blot. Immunofluorescence staining was used to analyze the co‐localization of Budding uninhibited by benzimidazole 1 (BUB1) and Kinesin family member 20A (KIF20A) in CC cells. Fe^2+^, MDA, and GSH levels were measured by commercial kits. Cell viability was determined by CCK‐8. The molecular interactions were analyzed by RIP or Co‐IP. MeRIP was adopted to measure the m^5^C level of KIF20A. We found that hypoxia facilitated ferroptosis resistance in CC cells. Hypoxia led to the upregulation of KIF20A, and KIF20A knockdown weakened hypoxia‐mediated ferroptosis resistance in CC cells. Mechanistically, Aly/REF export factor (ALYREF) stabilized KIF20A mRNA stability via m^5^C modification. In addition, KIF20A upregulated BUB1 in CC cells by directly interacting with BUB1. Rescue experiments indicated that BUB1 overexpression partially reversed the inhibitory effect of KIF20A knockdown on hypoxia‐mediated ferroptosis resistance in CC cells. In conclusion, hypoxia triggers ALYREF‐mediated m^5^C methylation of KIF20A to activate the KIF20A/BUB1 axis, thereby inducing ferroptosis resistance in CC cells.

**Abbreviations**
m^5^C: 5‐methylcytosine
ActD: actinomycin D
ALYREF: Aly/REF export factor
ANOVA: analysis of variance
ATCC: American type culture collection
BUB1: budding uninhibited by benzimidazoles 1
CCK‐8: cell counting kit‐8
CC: cervical cancer
Co‐IP: coimmunoprecipitation
GAPDH: reduced glyceraldehyde‐phosphate dehydrogenase
GSH: glutathion
HIF1α: hypoxia‐induced factor 1α
KIF20A: Kinesin family member 20A
MDA: malondialdehyde
Me‐RIP: methylated RNA immunoprecipitation
MUT: mutant
PVDF: polyvinylidene fluoride
RT‐qPCR: real‐time quantitative polymerase chain reaction
RIP: RNA immunoprecipitation
SD: standard deviation
SDS‐PAGE: sodium dodecyl sulfate polyacrylamide gel electrophoresis
sh‐KIF20A: short hairpin RNA for KIF20A
TCGA: the Cancer Genome Atlas
WT: wild‐type

## 1 Introduction

Cervical cancer (CC) is the most common gynecological cancer in women, with 604,127 new cases and 341,831 deaths worldwide annually [1, 2]. Currently, surgical removal in combination with chemoradiotherapy is the main treatment for early CC; however, there is still no effective therapy for advanced CC with metastasis [3]. Therefore, it is urgent to uncover the pathological mechanisms for its occurrence and development, which is essential to develop new treatment strategies for CC.

Ferroptosis is a novel type of programmed cell death, characterized by iron overload and accumulation of lipid peroxides [4]. Ferroptosis is a key contributor to the progression of malignancies, including CC. For example, ATF2‐mediated inhibition of ferroptosis promoted CC cell survival [5]. As reported, the uncontrolled proliferation of solid tumors can result in a hypoxic condition [6]. It has been confirmed that hypoxia caused chemotherapy resistance of CC cells [7]. Notably, hypoxia‐induced factor 1α (HIF1α) could transcriptionally regulate the expressions of transferrin receptor 1 and divalent metal transporter 1 [8], indicating the regulation of hypoxia in ferroptosis. So far, it has been suggested that hypoxic condition may lead to ferroptosis resistance in CC cells, whereas its underlying mechanisms remain unclear.

Kinesin family member 20A (KIF20A) belongs to the kinesin‐6 superfamily that can bind to guanosine triphosphate to affect cell cycle mitosis [9]. Zhang et al. reported that high expression of KIF20A was associated with poor overall survival and tumor progression in cervical squamous cell carcinoma [10]. In addition, KIF20A upregulation accelerated the proliferation and invasion of CC cells [11, 12]. Notably, KIF20A knockdown could trigger ferroptosis of cancer cells [13]. Wan et al. documented that positive expression of KIF20A, a hypoxia‐related biomarker, was associated with the malignant development of glioma [14]. However, the involvement of KIF20A in hypoxia‐mediated ferroptosis resistance in CC cells remains obscure, which deserves to be further investigated.

5‐methylcytosine (m5C) is an important RNA modification in coding and non‐coding RNAs [15]. Aly/REF export factor (ALYREF) is a reader of m5C, which recognizes m5C‐modified mRNAs [16]. ALYREF‐mediated m5C of NDRG1 conferred radioresistance of CC cells [17]. Moreover, ALYREF enhanced resistance to ferroptosis via modulation of m5C‐dependent PKM2 stabilization in intrahepatic cholangiocarcinoma [18]. In addition, RM2Target database predicted a potential interaction between KIF20A and ALYREF. Thus, we speculated that ALYREF might regulate m5C of KIF20A in hypoxia‐mediated ferroptosis resistance of CC cells.

Budding uninhibited by benzimidazole 1 (BUB1) is a conserved mitotic checkpoint serine/threonine kinase, which has been recognized as an oncogene in cancers [19, 20]. It has been reported that upregulation of BUB1 was involved in hypoxia‐induced epithelial‐mesenchymal transition of lung cancer cells [21]. Moreover, BUB1 was demonstrated to repress ferroptosis of pancreatic cancer cells, thus leading to gemcitabine resistance [22]. Interestingly, the ENCORI database predicted an interaction between KIF20A and BUB1. So far, whether KIF20A affected hypoxia‐mediated ferroptosis resistance of CC cells via interaction with BUB1 has not been clarified.

Based on the above evidence, we speculated that hypoxia might upregulate KIF20A by promoting ALYREF‐mediated m^5^C methylation and subsequently interact with BUB1; thereby leading to ferroptosis resistance of CC cells. Our research might provide a theoretical basis for the development of novel treatment strategies for CC.

## 2 Materials and Methods

### 2.1 Cell Culture and Treatment

Human CC cell lines (SiHa cells, CaSki cells, C‐33A cells, and MS751 cells) and 293 T cells were purchased from American Type Culture Collection (ATCC, VA, USA). All cells were grown in DMEM (Gibco, MD, USA) containing 10% FBS at 37°C with 5% CO_2_. To induce hypoxia, the cells were maintained in a CO₂ incubator (3110 Series II, Thermo Fisher, MA, USA) with 1% O_2_, 5% CO_2_, and 95% N_2_ for 0.5, 1, 2, 4, and 12 h. The cells in the normoxia group were cultured under normal conditions. After 2 h of culture under hypoxia or normoxia conditions, the cells were treated with 5 μmol/L Erastin (MCE, NJ, USA) or 1 μmol/L ferrostatin‐1 (MCE) for 24 h [23, 24, 25].

### 2.2 Cell Transfection

The overexpression plasmid (pcDNA3.1‐BUB1) and the short hairpin RNA for KIF20A (sh‐KIF20A, sequences: 5′‐CAGGAGGTTAAAGCTAAAT‐3′ and sh‐ALYREF, sequences: 5′‐AGAGTTCCTGAATATCGGC‐3′) as well as their negative controls were purchased from GenePharma (Shanghai, China). Cells were seeded into 6‐well plates (5 × 10^5^ cells per well) and transfected with the above plasmids (100 nM) or shRNAs (1 μg) using Lipofectamine 3000 (Invitrogen, CA, USA) according to the standard protocol. The next day, the transfected cells were subjected to hypoxia for 2 h, followed by treatment with 5 μmol/L Erastin or 1 μmol/L ferrostatin‐1 for 24 h. At 48 h post‐transfection, the cells were collected for subsequent experiments.

### 2.3 Cell Counting Kit‐8 (CCK‐8)

Cells were planted into 96‐well plates (2000 cells per well). After cells were attached, the cells were incubated with 10 μL CCK‐8 solution (Yeason, Shanghai, China) for 3 h at 37°C. The absorbance at 450 nm was measured on a microplate reader (Thermo Fisher, MA, USA).

### 2.4 The Measurement of Iron, Malondialdehyde (MDA), and Glutathione (GSH) Levels

Caski, MS751, and C‐33A cells were seeded into 6‐well plates (2 × 10^5^ cells per well) and subjected to hypoxia stimulation (0–12 h). Iron, MDA, and GSH levels in cells were examined using the iron assay kit (Abcam, Cambridge, UK, ab83366), MDA assay kit (Abcam, ab118970), and GSH assay kit (Abcam, ab112132), respectively, strictly according to the manufacturer's manuals.

### 2.5 Real‐Time Quantitative Polymerase Chain Reaction (RT‐qPCR)

CaSki and C‐33A cells were seeded into 6‐well plates (2 × 10^5^ cells per well) and subjected to hypoxia stimulation (0–12 h). The total RNA was extracted using TRIzol (Thermo Fisher). The cDNA was synthesized using the HiFiScript cDNA synthesis kit (Toyobo, Tokyo, Japan). RT‐qPCR assay was performed using the PowerTrack SYBR Green Master Mix (Thermo Fisher). In brief, the samples were first denatured at 95°C for 15 min, followed by 40 cycles of denaturation at 95°C for 15 s, annealing at 58°C for 15 s, and elongation at 72°C for 20 s. Reduced glyceraldehyde‐phosphate dehydrogenase (GAPDH) was used as the housekeeper gene. The data were analyzed with the 2^−ΔΔCT^ method. The primer sequences are as follows: (5′‐3′): KIF20A (F): TGCTGTCCGATGACGATGTC; KIF20A (R): AGGTTCTTGCGT

ACCACAGAC. ALYREF (F): GCAGGCCAAAACAACTTCCC; ALYREF (R): AGTTCCTGAATATCGGCGTCT. BUB1 (F): TGGGAAAGATACATACAGTGGGT; BUB1 (R): AGGGGATGACAGGGTTCCAAT. GAPDH (F): AGGTCGGTGTGAAC

GGATTTG; GAPDH (R): GGGGTCGTTGATGGCAACA

### 2.6 Western Blot

The proteins were extracted using the RIPA lysis buffer (Beyotime, Shanghai, China). The protein concentration was determined using the BCA kit (Beyotime), and the total protein samples (20 μg) were separated by 10% sodium dodecyl sulfate polyacrylamide gel electrophoresis (SDS‐PAGE) and transferred onto the polyvinylidene fluoride (PVDF) membranes (Millipore, MA, USA). The membranes were blocked for 1 h with 5% skimmed milk and then incubated overnight with primary antibodies against KIF20A (ab70791), ALYREF (ab202894), BUB1 (ab195268), and β‐actin (ab8226). The membranes were then incubated with the secondary antibody (ab7090) for 1 h. The bands were visualized using the ECL kit (Beyotime), and Image J software was used to quantify the gray values of the protein bands. All antibodies were obtained from Abcam (Cambridge, UK) and diluted according to the instructions.

### 2.7 Bioinformatics Analysis

The sequences of the promoter region of the ALYREF gene (2000 bp upstream and 100 bp downstream of transcription start site) were obtained from the NCBI database (chr17: 81891488–81,893,587). The binding of HIF‐1α to the ALYREF promoter region (from −2000 to +100 bp) was analyzed using the JASPAR database (http://jaspar.genereg.net/).

### 2.8 Dual Luciferase Reporter Assay

The wild‐type (WT) sequences containing binding sites (GCACGTGCCT) of hypoxia‐inducible factor—1α (HIF‐1α) to ALYREF promoter located at positions from −1085 to −1076, or the deletion mutant (MUT) sequences were synthesized by GenePharma and inserted downstream of the Renilla luciferase in psicheck2 luciferase vector (Promega, WI, USA) using SalI/XhoI and NotI digestion. 293 T cells (5 × 10^5^) were seeded into 24‐well plates and transfected with ALYREF‐WT or ALYREF‐MUT plasmids by Lipofectamine 3000. After transfection for 48 h, the luciferase activities were determined by the Dual Luciferase Assay kit (Promega).

### 2.9 RNA Binding Protein Immunoprecipitation (RIP) Assay

CaSki and C‐33A cells were seeded into 6‐well plates (5 × 10^5^ cells per well). RIP assay was performed using the MagnaRIP RNA‐binding Immunoprecipitation Kit (Millipore). The cell lysates (150 μg protein) were incubated with 5 μg ALYREF antibody (Abcam, 1:40, ab202894) or IgG antibody (Abcam, 1:100, ab109489), followed by incubation with protein A/G magnetic beads for 1 h. RNAs were purified from the mRNA‐bead‐antibody complex and subjected to RT‐qPCR and agarose gel electrophoresis analysis. The primer sequences are as follows: (5′–3′): KIF20A (F): TGCTGTCCGATGACGATGTC; KIF20A (R): AGGTTCTTGCGTACCACAGAC.

### 2.10 Methylated RNA Immunoprecipitation (Me‐RIP) Assay

Me‐RIP assay was performed using the Magna MeRIP Kit (Millipore). The total RNAs extracted from cells were fragmented into 100–200 bp. The RNAs were incubated with 10 μg m^5^C antibody (biorbyt, Hubei, China, 1:1000, orb1150382) and then incubated with protein A/G magnetic beads for 1 h. RNAs were purified from the mRNA‐bead‐antibody complex. The abundance of m^5^C in purified RNAs (150 μg) was detected by RT‐qPCR.

### 2.11 Co‐Immunoprecipitation (Co‐IP)

CaSki and C‐33A cells (5 × 10^5^ cells per well in 6‐well plates) were collected after seeding for 24 h and lysed in RIPA lysis buffer (Beyotime) containing 50 mM Tris (pH 7.4), 150 mM NaCl, 1% Triton X‐100, 1% sodium deoxycholate, 0.1% SDS, sodium orthovanadate, sodium fluoride, EDTA, and protease and phosphatase inhibitors. The cell lysates (500 μg) were incubated overnight with 2 μg IgG (Abcam, ab172730), KIF20A (Abcam, ab70791) or BUB1 (Thermo Fisher, PA5‐144224) antibody at 4°C. The Protein A‐Sepharose CL‐4B beads (30 μL, Sigma‐Aldrich) were washed twice with 200 μL PBS buffer and then incubated with the cell lysates and antibody for 3 h. Following the IP, the beads were centrifuged at 1000 g at 4°C for 2 min and rinsed with lysis buffer five times, followed by boiling for 5 min at 100°C. Finally, the bound proteins were analyzed using Western blot.

### 2.12 RNA Stability Assay

Cells were seeded in 6‐well plates (5 × 10^5^ cells per well). After transfection for 48 h, the cells were treated with 5 μg/mL actinomycin D (ActD, Sigma‐Aldrich) for 0, 2, 4, 6, and 8 h. After incubation for the specified time points, the cells were collected, and the mRNA level of KIF20A was quantified by RT‐qPCR.

### 2.13 Immunofluorescence Staining

CaSki and C‐33A cells (5 × 10^5^ cells per well in 6‐well plates) were fixed with 4% paraformaldehyde for 10 min and then sealed with goat serum for 30 min. Cells were then incubated with the primary antibodies against BUB1 (Abcam, 1:100, ab195268) and KIF20A (Santa Cruz, TX, USA, 1:50, sc‐374,508) overnight at 4°C. Cells were subsequently incubated with the corresponding secondary antibody (Abcam, 1:200, ab150077 or ab150115) for 1 h. DAPI (Sangon, Shanghai, China) was employed to stain the nuclei. The cells were sealed with the sealing liquid containing anti‐fluorescence quenching reagent and photographed under a fluorescence microscope (Olympus, Tokyo, Japan).

### 2.14 Data Analysis

All data from three independent experiments are expressed as means ± standard deviation (SD) and analyzed by SPSS 19.0. The differences between two groups were analyzed by Student's *t*‐tests. One‐way analysis of variance (ANOVA) was performed to assess the differences among multiple groups. The *p* values less than 0.05 were considered statistical significance.

## 3 Results

### 3.1 Hypoxia Repressed CC Cell Viability and Induced Ferroptosis Resistance

**FIGURE 1:**
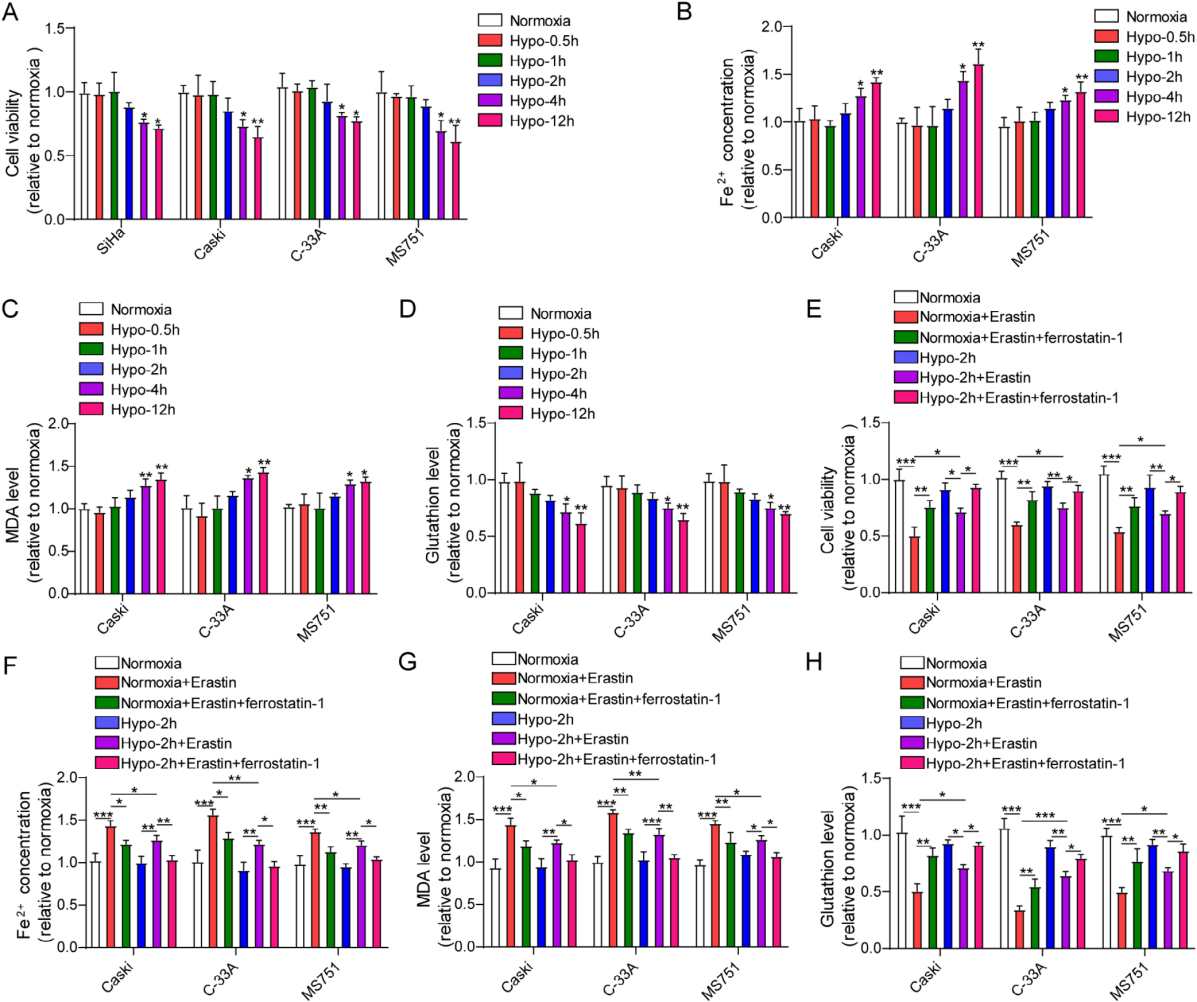
Hypoxia repressed CC cell viability and induced ferroptosis resistance. (A) SiHa, Caski, MS751, and C‐33A cells were subjected to hypoxia stimulation (0–12 h), and cell viability was determined by CCK‐8 assay on a microplate reader. (B–D) Caski, MS751, and C‐33A cells were subjected to hypoxia stimulation (0–12 h), and Fe^2+^, MDA, and GSH levels in cells were measured by the commercial kits on a microplate reader. SiHa, Caski, MS751, and C‐33A cells were cultured under normoxic conditions or hypoxic conditions for 2 h, followed by treatment with 5 μmol/L Erastin (ferroptosis inducer) and 1 μmol/L ferrostatin‐1 (ferroptosis inhibitor) for 24 h. (E) CCK‐8 assay was employed to assess cell viability on a microplate reader. (F–H) Fe^2+^, MDA, and GSH levels in cells were measured by the kits on a microplate reader. The measurement data are presented as mean ± SD. All data were obtained from at least three replicate experiments. *p < 0.05, **p < 0.01, ***p < 0.001.

First, we evaluated the influence of hypoxia on CC cell viability. It was observed that hypoxia did not affect the viability of CC cells (SiHa cells, CaSki cells, C‐33A cells, and MS751 cells) at 0.5–2 h time points, while significantly reducing CC cell viability at 4 and 12 h time points (Figure 1A). Subsequently, the regulation of hypoxia in ferroptosis of CC cells was evaluated. Our results demonstrated that hypoxia exposure for 0.5–2 h did not affect intracellular iron, MDA, or GSH levels compared to normoxia, whereas prolonged hypoxia for 4–12 h remarkably increased the levels of iron and MDA and reduced GSH level in CaSki cells, C‐33A cells, and MS751 cells (Figure 1B–D). These results showed that 2‐h hypoxia did not affect ferroptosis in CC cells. Therefore, Erastin (ferroptosis inducer) or ferrostatin‐1 (ferroptosis inhibitor) was added after hypoxia for 2 h. Under normoxic conditions, iron and MDA levels were increased, but CC cell viability and GSH level were lowered by Erastin, which could be partly reversed by ferrostatin‐1 (Figure 1E–H). However, the above changes were weakened under hypoxic condition (Figure 1F–H). These findings suggested that hypoxia for 2 h caused ferroptosis resistance in CC cells.

### 3.2 Hypoxia Upregulated KIF20A in CC Cells

**FIGURE 2:**
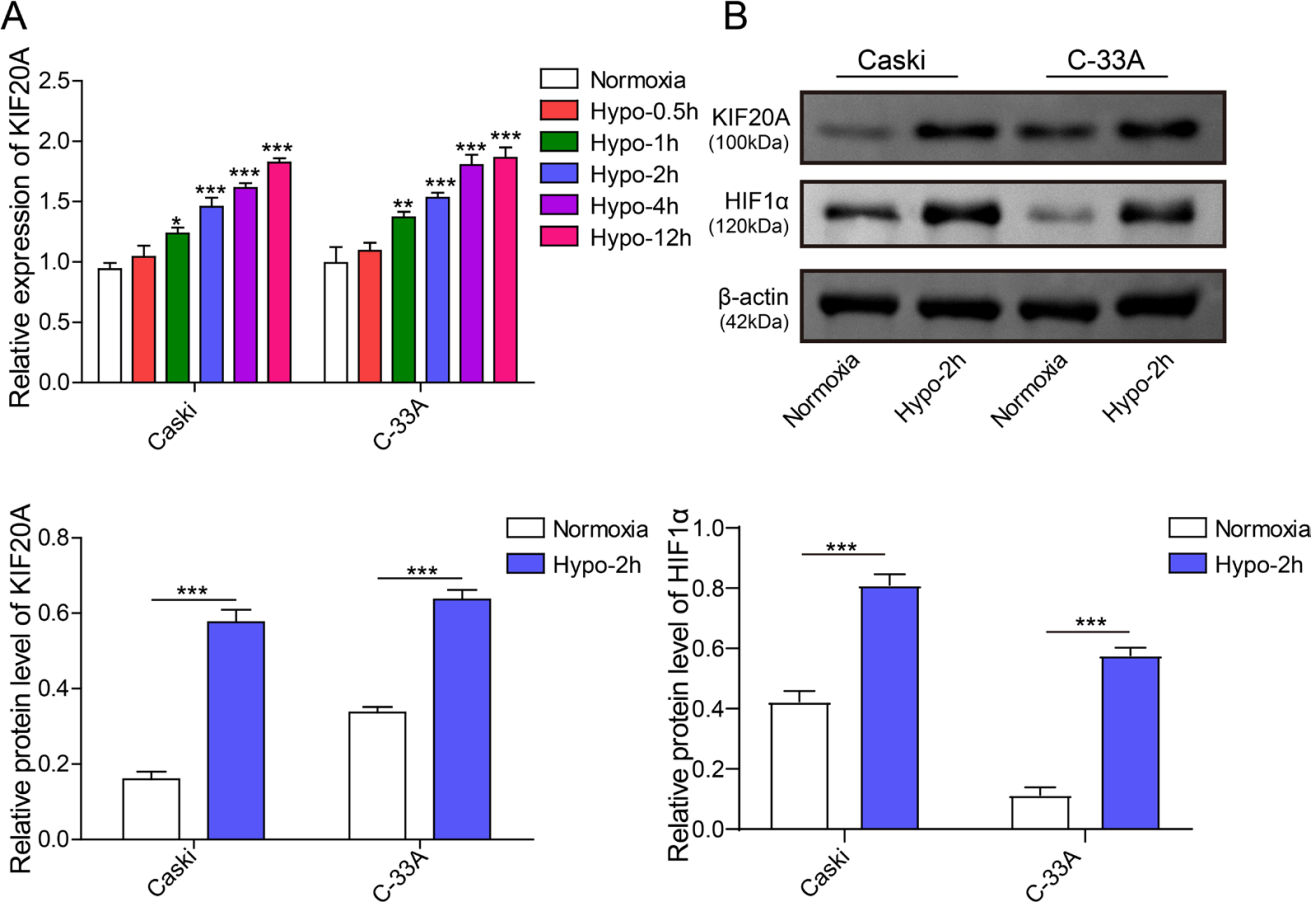
Hypoxia enhanced KIF20A expression in CC cells. (A) CaSki and C‐33A cells were subjected to hypoxia stimulation (0–12 h), and KIF20A mRNA level in cells was determined using RT‐qPCR. (B) CaSki and C‐33A cells were subjected to 2 h‐hypoxia stimulation, and western blot was employed to detect HIF‐1α and KIF20A protein levels. The measurement data are presented as mean ± SD. All data were obtained from at least three replicate experiments. *p < 0.05, **p < 0.01, ***p < 0.001.

Next, the potential mechanism responsible for hypoxia‐induced ferroptosis resistance in CC cells was explored. As analyzed by the Cancer Genome Atlas (TCGA) database, KIF20A was widely overexpressed in various types of human tumors (Figure S1A). In addition, the TCGA database showed that KIF20A was highly expressed in CC tissues as compared with the normal cervical epithelial tissues (Figure S1B). Notably, exposure to hypoxia strikingly elevated KIF20A mRNA level in CC cells in a time‐dependent manner (Figure 2A). As expected, the expression of HIF‐1α in CaSki and C‐33A cells under hypoxic conditions showed a rising trend (Figure 2B). Moreover, 2 h‐hypoxia also increased KIF20A protein level in CC cells (Figure 2B). Collectively, KIF20A was upregulated by hypoxia in CC cells.

### 3.3 KIF20A Knockdown Weakened Hypoxia‐Mediated Ferroptosis Resistance in CC Cells

**FIGURE 3:**
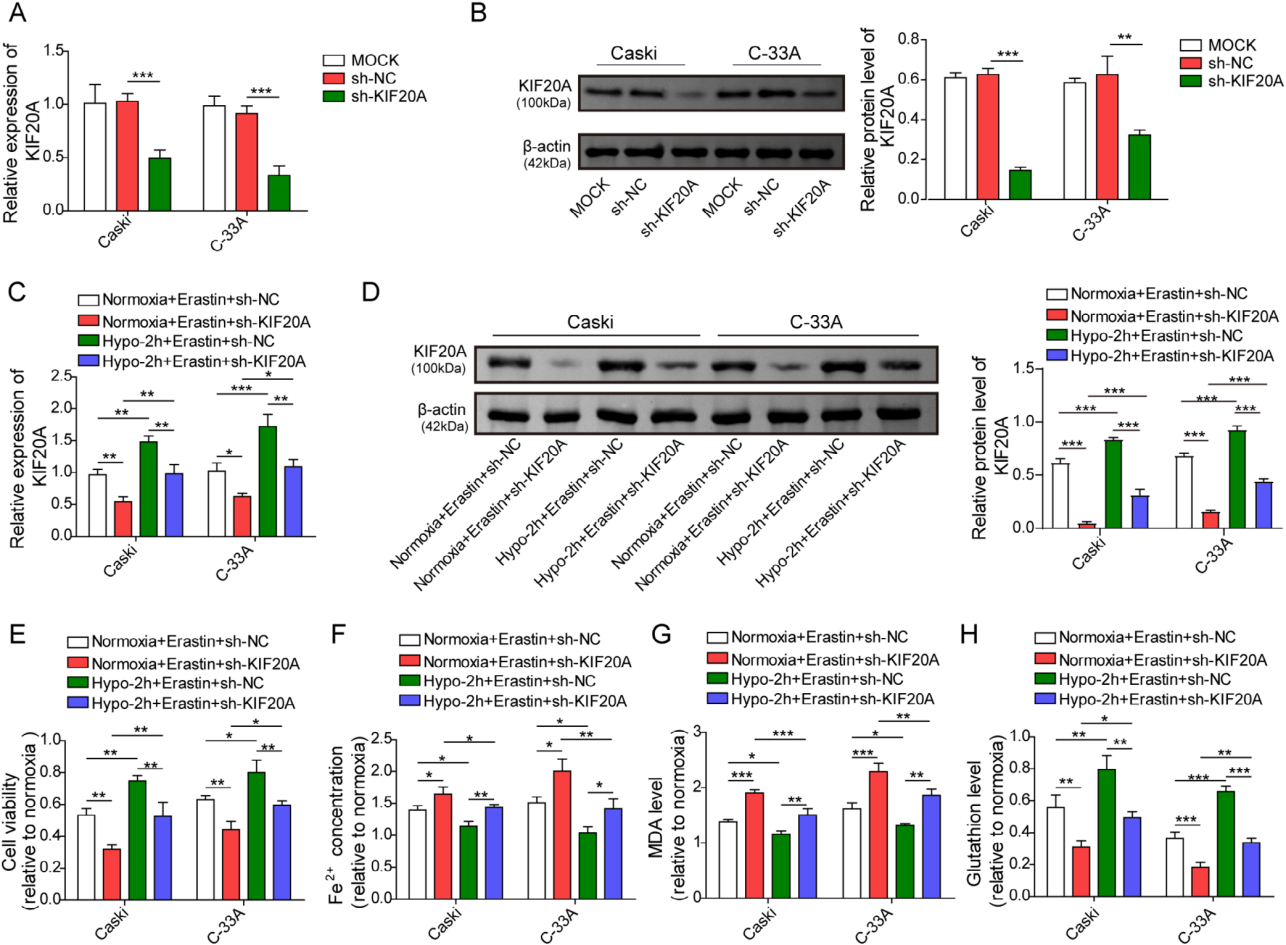
KIF20A knockdown weakened hypoxia‐mediated ferroptosis resistance in CC cells. CaSki and C‐33A cells were transfected with 1 μg sh‐NC or sh‐KIF20A, and the mRNA and protein levels of KIF20A in cells were determined by RT‐qPCR (A) and western blot (B). CaSki and C‐33A cells were transfected with 1 μg sh‐NC or sh‐KIF20A, and then subjected to hypoxia or normoxia for 2 h, followed by treatment with 5 μmol/L Erastin for 24 h. (C, D) RT‐qPCR and western blot were employed to measure KIF20A level in cells. (E) Cell viability was determined by CCK‐8 assay. (F–H) Fe^2+^, MDA, and GSH levels in cells were measured by the corresponding kits. The measurement data are presented as mean ± SD. All data were obtained from at least three replicate experiments. *p < 0.05, **p < 0.01, ***p < 0.001.

To investigate whether KIF20A was involved in hypoxia‐induced ferroptosis resistance in CC cells, KIF20A was silenced in CC cells by transfection with sh‐KIF20A. We observed that sh‐KIF20A transfection significantly reduced KIF20A mRNA (Figure 3A) and protein levels (Figure 3B) in CaSki and C‐33A cells. Furthermore, sh‐KIF20A transfection significantly reduced KIF20A mRNA and protein expression in Erastin‐treated CC cells under both normoxic and hypoxic conditions (Figure 3C,D). Moreover, 2‐h hypoxia led to an elevation in cell viability and GSH level, while reducing iron and MDA levels in Erastin‐treated CC cells, as compared with the normoxic group (Figure 3E–H). As expected, knockdown of KIF20A exhibited the opposite roles and reversed 2‐h hypoxia‐induced the above changes (Figure 3E–H). Taken together, hypoxia‐induced ferroptosis resistance in CC cells was attenuated by KIF20A deficiency.

### 3.4 ALYREF Directly Bound to KIF20A to Enhance Its mRNA Stability in an m^5^C‐Dependent Manner

**FIGURE 4:**
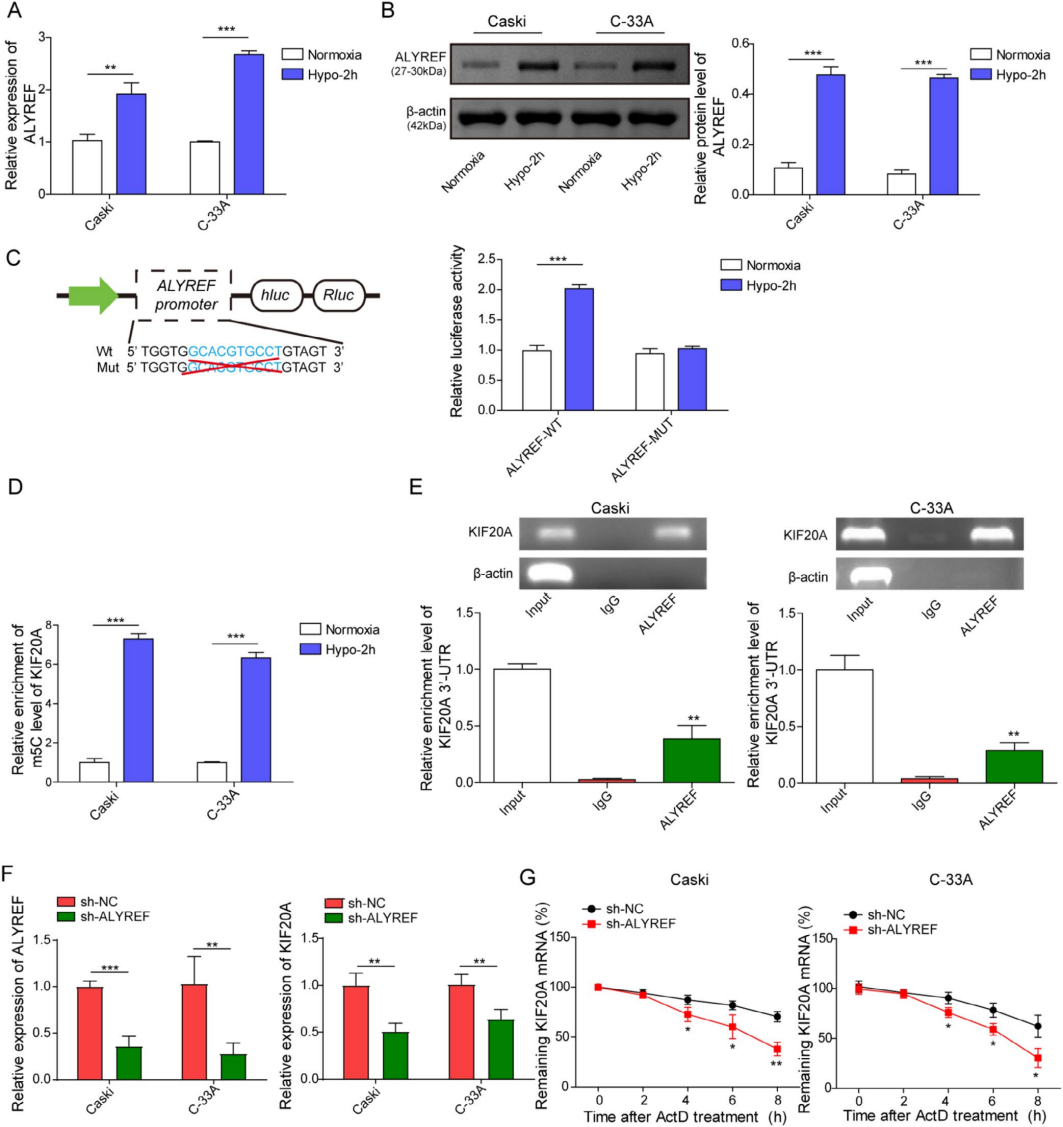
ALYREF directly bound to KIF20A mRNA to increase its mRNA stability in an m^5^C‐dependent manner. (A, B) CaSki and C‐33A cells were subjected to 2 h‐hypoxia or normoxia stimulation, and ALYREF mRNA and protein level in cells were assessed using RT‐qPCR and western blot. (C) The relative luciferase activity in 293 T cells transfected with 100 nM ALYREF‐WT or ALYREF‐MUT after 2 h‐hypoxia or normoxia stimulation was measured. (D) MeRIP assay measured the m^5^C level of KIF20A in CC cells after 2 h‐hypoxia or normoxia stimulation. (E) The interaction between KIF20A and ALYREF in CaSki and C‐33A cells was analyzed by RIP assay. (F) The mRNA levels of ALYREF and KIF20A in CC cells after 1 μg sh‐NC or sh‐ALYREF transfection for 48 h were detected using RT‐qPCR. (G) KIF20A mRNA stability in CC cells after 1 μg sh‐NC or sh‐ALYREF transfection for 48 h was determined using mRNA stability assay. The measurement data are presented as mean ± SD. All data were obtained from at least three replicate experiments. *p < 0.05, **p < 0.01, ***p < 0.001.

To further elucidate the upstream regulatory mechanism of KIF20A, we focused on m^5^C, an important post‐transcriptional modification. RM2Target database predicted that KIF20A could be methylated by ALYREF‐mediated m^5^C (Figure S1C). As revealed in Figure 4A,B, hypoxia stimulation evidently enhanced ALYREF mRNA and protein levels in CaSki and C‐33A cells. JASPAR database predicted HIF‐1α as the potential transcription factor for ALYREF, and the core HIF‐1α binding motif (ACGTG) is located at positions −1083 to −1079 of the ALYREF promoter (Figure S1D). Subsequently, dual luciferase reporter assay showed that 2 h‐hypoxia significantly increased the luciferase activity of ALYREF‐WT, but not affected that of ALYREF‐MUT in 293 T cells (Figure 4C). In addition, the m^5^C level of KIF20A was increased by 2 h‐hypoxia in CC cells (Figure 4D). Moreover, RIP assay showed that ALYREF directly bound to the 3′‐UTR of KIF20A in CC cells (Figure 4E). Besides, ALYREF silencing strikingly reduced the mRNA level and mRNA stability of KIF20A in CC cells (Figure 4F,G). In summary, hypoxia‐mediated upregulation of ALYREF enhanced the mRNA stability of KIF20A in CC cells in an m^5^C‐dependent manner.

### 3.5 KIF20A Was Positively Correlated and Co‐Localized With BUB1 in the Nuclei of CC Cells

**FIGURE 5:**
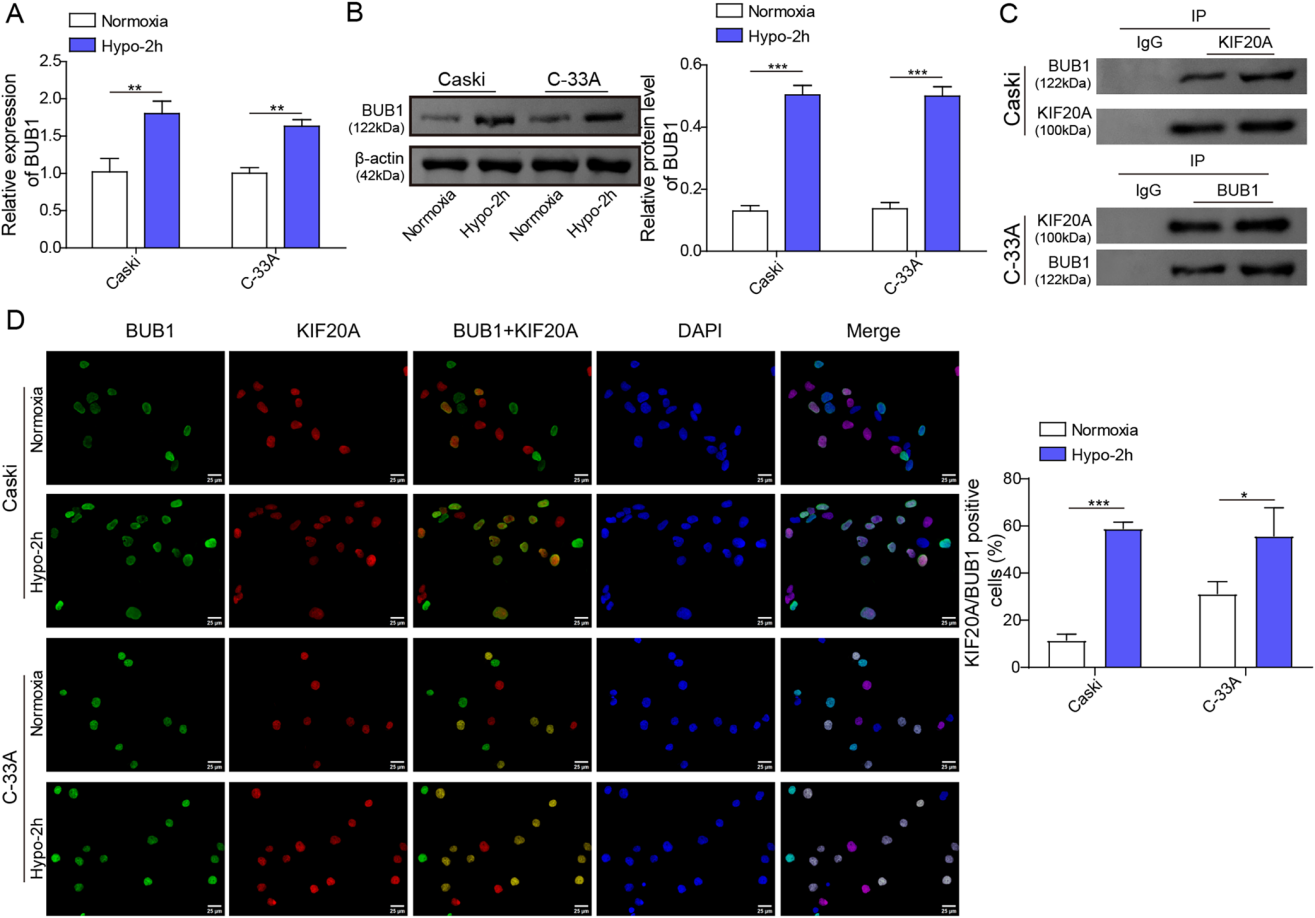
KIF20A was positively correlated and co‐localized with BUB1 in the nuclei of CC cells. (A, B) CaSki and C‐33A cells were subjected to 2 h‐hypoxia or normoxia stimulation, and BUB1 mRNA and protein levels in cells were assessed using RT‐qPCR and western blot. (C) Co‐IP assay was performed to analyze the interaction between KIF20A and BUB1 in CaSki and C‐33A cells. (D) Immunofluorescence staining was used to analyze the cellular co‐localization of BUB1 and KIF20A in CaSki and C‐33A cells. Scale bar, 25 μm. The measurement data are presented as mean ± SD. All data were obtained from at least three replicate experiments. *p* < 0.05, **p < 0.01, ***p < 0.001.

We further explored the downstream mechanism of KIF20A through which regulated hypoxia‐mediated ferroptosis resistance in CC cells. As displayed in Figure S1E, pan‐cancer co‐expression analysis by the ENCORI database revealed that there was a positive correlation between KIF20A and BUB1 mRNA levels in CC tissues. Furthermore, the TCGA database indicated that BUB1 was highly expressed in CC tissues in comparison with normal cervical epithelial tissues (Figure S1F). In addition, RT‐qPCR and western blot demonstrated that hypoxia stimulation dramatically elevated BUB1 expression in CaSki and C‐33A cells (Figure 5A,B). As analyzed by the Biogrid database, there was a potential binding relationship between KIF20A and BUB1 (Figure S1G). Besides, the Co‐IP assay indicated that KIF20A protein directly interacted with BUB1 protein (Figure 5C). Moreover, immunofluorescent staining showed that KIF20A and BUB1 were co‐localized in the nuclei of CC cells (Figure 5D). Collectively, KIF20A could directly interact with BUB1 in CC cells.

### 3.6 KIF20A/BUB1 Axis Participated in Hypoxia‐Mediated Ferroptosis Resistance in CC Cells

**FIGURE 6:**
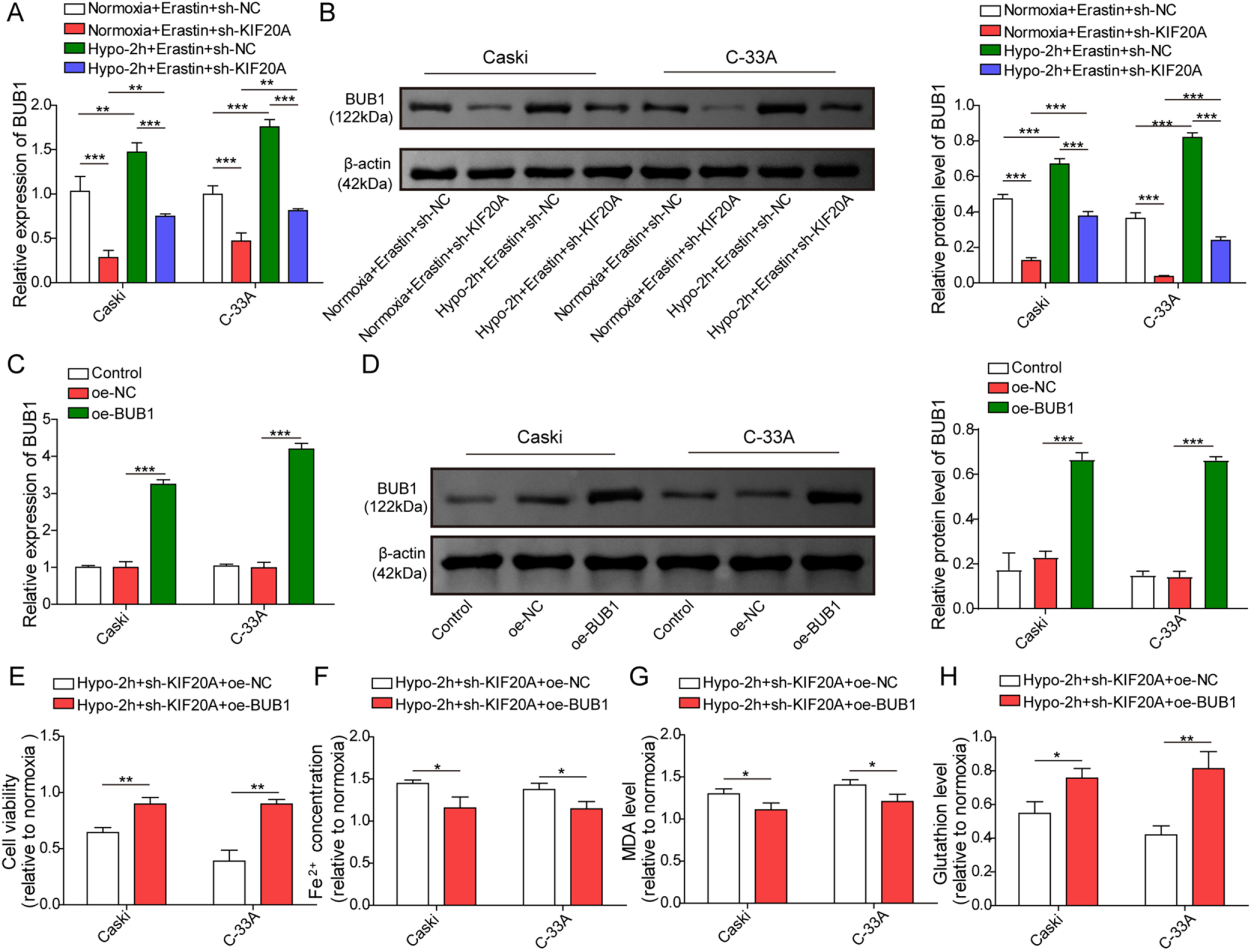
KIF20A/BUB1 axis was involved in hypoxia‐mediated ferroptosis resistance in CC cells. (A, B) CC cells were transfected with 1 μg sh‐NC or sh‐KIF20A, and then subjected to hypoxia or normoxia for 2 h, followed by treatment with 5 μmol/L Erastin for 24 h. BUB1 mRNA and protein levels in cells were determined by RT‐qPCR and western blot. CC cells were transfected with 1 μg sh‐KIF20A and 100 nM pcDNA3.1‐BUB1, and then subjected to hypoxia for 2 h. (C, D) BUB1 mRNA and protein levels in cells were detected using RT‐qPCR and western blot. (E) CCK‐8 assay was employed to assess cell viability. (F–H) Fe^2+^, MDA, and GSH levels in cells were measured by the kits. The measurement data are presented as mean ± SD. All data were obtained from at least three replicate experiments. *p < 0.05, **p < 0.01, ***p < 0.001.

Finally, we examined whether the KIF20A/BUB1 axis regulated hypoxia‐mediated ferroptosis resistance in CC cells. As shown in Figure 6A,B, sh‐KIF20A transfection effectively reduced BUB1 expression in CaSki and C‐33A cells under both normoxic and hypoxic conditions (Figure 6A,B). To study the involvement of the KIF20A/BUB1 axis in the modulation of hypoxia‐mediated ferroptosis resistance in CC cells, hypoxia‐exposed CC cells were co‐transfected with sh‐KIF20A and pcDNA3.1‐BUB1. We found that pcDNA3.1‐BUB1 transfection significantly enhanced BUB1 expression in CaSki and C‐33A cells (Figure 6C,D). In addition, BUB1 overexpression enhanced the viability of KIF20A‐silenced CC cells under hypoxic conditions (Figure 6E). Furthermore, BUB1 overexpression markedly reduced Fe^2+^ and MDA levels, while increasing GSH levels in KIF20A deficient CC cells after exposure to hypoxia (Figure 6F–H). To sum up, BUB1 was involved in KIF20A‐mediated ferroptosis resistance in CC cells.

## 4 Discussion

Our primary findings revealed that hypoxia upregulated BUB1 in CC cells by increasing KIF20A expression through promoting ALYREF‐mediated m^5^C methylation of KIF20A, thereby inducing ferroptosis resistance in CC cells. CC is the most common gynecological tumor worldwide. The current therapies for CC mainly include radiotherapy, chemotherapy, and surgery. However, the prognosis of advanced CC patients is still poor, mainly due to recurrence, metastasis, and drug resistance [26]. A deeper understanding of the pathogenesis of CC may help to develop novel and effective treatments for CC. Our results provide theoretical evidence for the ALYREF/KIF20A/BUB1 axis as potential therapeutic targets for CC.

Hypoxia refers to a decrease in oxygen level at the cellular or tissue level, which is an outstanding characteristic of tumors [27]. As previously reported, hypoxia could result in the resistance of pancreatic cancer cells to ferroptosis [28]. In addition, hypoxia repressed ferroptosis in liver cancer cells [29]. Consistent with the previous studies, our results showed that 2 h‐hypoxia stimulation weakened Erastin‐induced ferroptosis of CC cells, indicating the induction of ferroptosis resistance in CC cells. Notably, KIF20A was upregulated in CC, which promoted CC cell proliferation and invasion [11]. Moreover, a previous study showed that KIF20A expression was significantly increased in lung cells in response to hypoxia stimulation [30]. Besides, inhibition of KIF20A could induce ferroptosis in colorectal cancer cells [13]. Nevertheless, whether KIF20A modulated hypoxia‐induced ferroptosis resistance in CC cells remains unclear. Our findings revealed that KIF20A was highly expressed in hypoxia‐stimulated CC cells. In addition, KIF20A silencing enhanced the ferroptosis sensitivity of CC cells under hypoxic conditions. Collectively, KIF20A upregulation contributed to hypoxia‐induced ferroptosis resistance in CC cells.

m^5^C is one of the epigenetic modifications, which plays a key role in the occurrence and development of various types of cancer [31]. ALYREF is an m^5^C reader protein that recognizes m^5^C‐modified mRNA and regulates its stabilization [32]. ALYREF has been recognized to act as an oncogene in multiple human malignant tumors [32, 33]. A previous study demonstrated that ALYREF reduced the radiosensitivity of CC cells by binding to m^5^C‐labeled NDRG1 mRNA to increase its stability [17]. Notably, ALYREF was activated by HIF‐1α, thereby promoting glycolysis and the development of bladder cancer [34], suggesting its role in hypoxia during tumorigenesis. Our findings for the first time reported that hypoxia significantly increased ALYREF expression in CC cells. Interestingly, our results also revealed that ALYREF‐mediated m^5^C modification stabilized KIF20A mRNA in CC cells, which was responsible for hypoxia‐induced ferroptosis resistance in CC cells.

BUB1 is a mitotic checkpoint serine/threonine kinase, which has been identified to be an oncogene in various types of cancers [35, 36]. A previous study demonstrated that BUB1 acted as a key contributor to CC development [37]. More importantly, it has been reported that hypoxia upregulated BUB1 to accelerate lung cancer development [21]. In line with the previous work, we found that hypoxia stimulation resulted in BUB1 upregulation in CC cells. Furthermore, KIF20A directly interacted with BUB1 to enhance BUB1 expression in CC cells. As expected, BUB1 overexpression weakened ferroptosis of KIF20A‐depleted CC cells under hypoxic conditions. In summary, hypoxia‐induced KIF20A upregulation was involved in ferroptosis resistance in CC cells by upregulating BUB1.

Taken together, our findings suggested that hypoxia stabilized KIF20A mRNA by promoting ALYREF‐mediated m^5^C methylation, which subsequently upregulated BUB1 to trigger ferroptosis resistance in CC cells. Our results indicate that targeting the ALYREF/KIF20A/BUB1 axis might be a potential treatment strategy for CC.

## Conflicts of Interest

The authors declare no conflicts of interest.

## Supporting information


**Data S1:** kjm270093‐sup‐0001‐supinfo.docx.

## Data Availability

The data that support the findings of this study are available from the corresponding author upon reasonable request.
