# Supplementary material for: Hypoxia Triggers ALYREF‐Mediated m5C Methylation of KIF20A to Activate KIF20A/BUB1 for Generating Ferroptosis Resistance in Cervical Cancer Cells
Source: Kaohsiung J Med Sci. 2025 Sep 7;42(1):e70093. doi: 10.1002/kjm2.70093 (PMC12782253; doi:10.1002/kjm2.70093)

**Supplementary materials**

**Supplementary figures and legends**

**
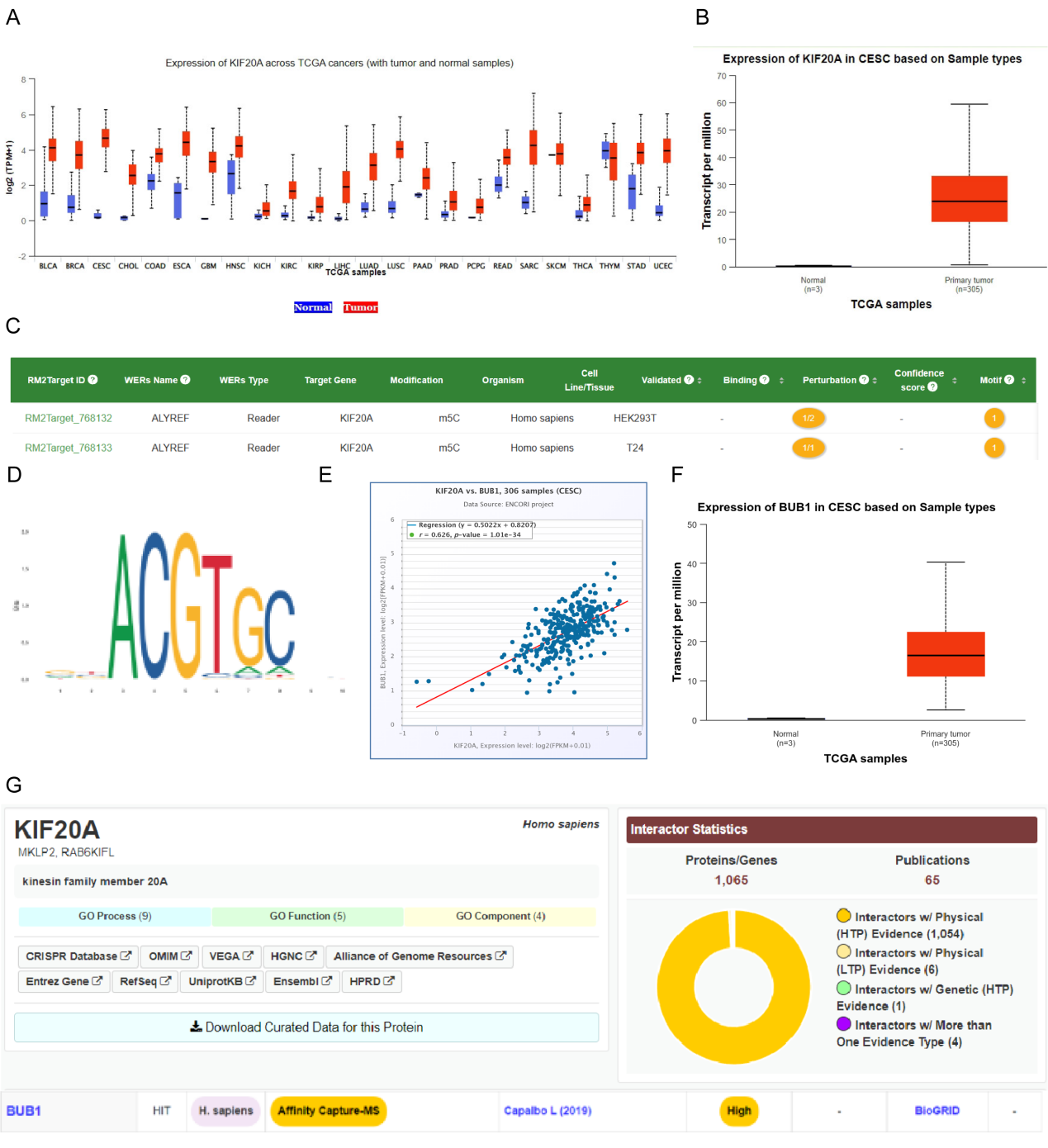
**

**Supplementary figure 1 Bioinformatics analysis.**

(A) KIF20A expression in different types of human tumors was predicted using the TCGA database. (B) KIF20A expression in CC tissues and normal cervical epithelial tissues was predicted using the TCGA database. (C) The interaction between KIF20A and ALYREF-mediated m^5^C process was predicted using the RM2Target database. (D) The consensus hypoxia response element for ALYREF was predicted using bioinformatic analysis. (E) ENCORI pan cancer co-expression analysis was employed to analyze the correlation between KIF20A expression and BUB1 expression in CC cells. (F) BUB1 expression in CC tissues and normal cervical epithelial tissues was predicted using the TCGA database. (G) The potential binding relationship between KIF20A and BUB1 was predicted using the Biogrid database.

**Supplementary data for original blot of WB.**


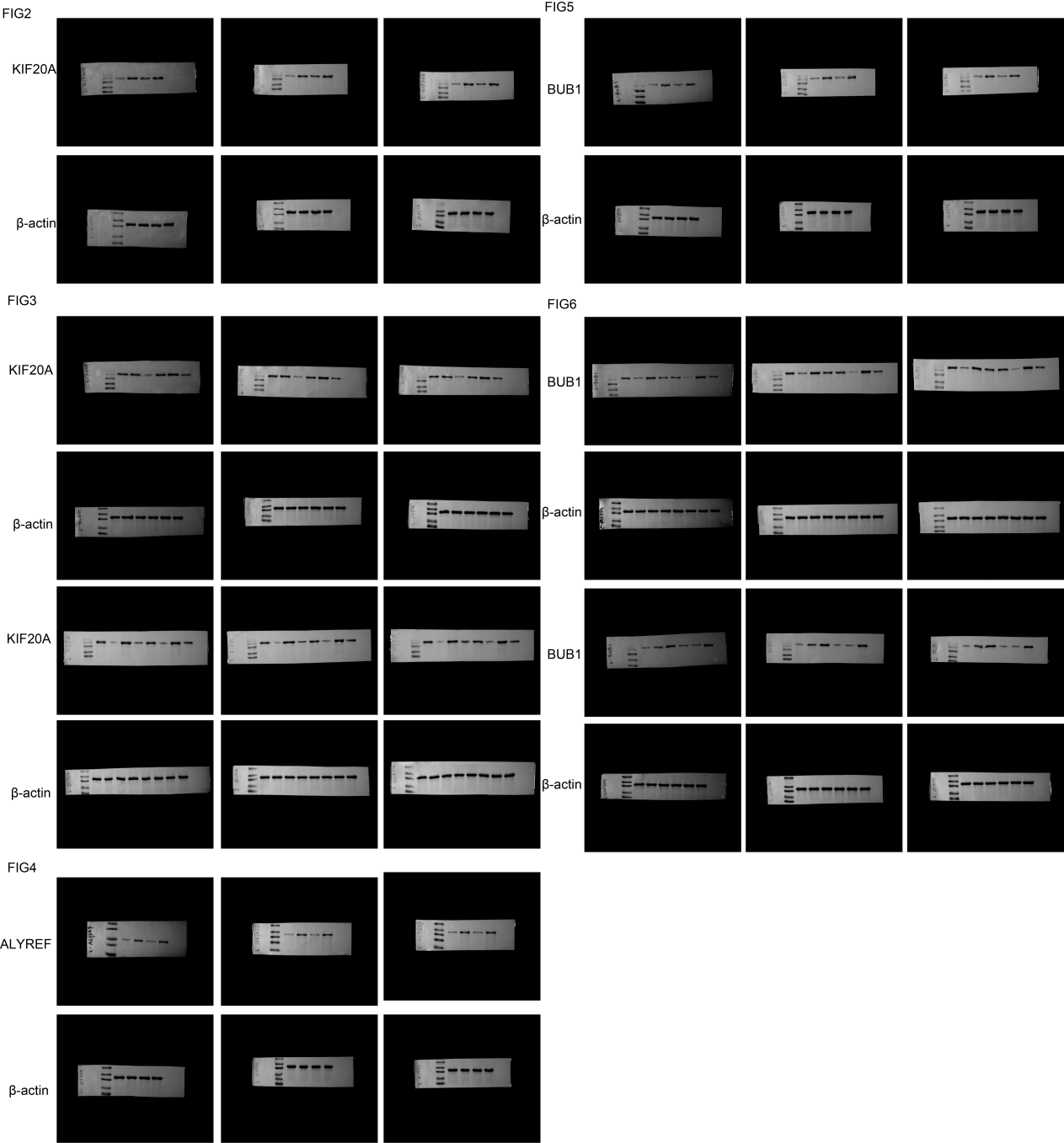

Supplement: Supplementary file 1 — Data S1: kjm270093‐sup‐0001‐supinfo.docx. [file KJM2-42-e70093-s001.docx]
